# Objective Assessment of the Cardiorespiratory Fitness Among Individuals With Lymphedema and Lipedema: A Systematic Review and Meta-Analysis

**DOI:** 10.1155/ijvm/8627520

**Published:** 2025-02-13

**Authors:** Amirparsa Vanaki, Amirhossein Fallah, Negin Rahimidanesh, Arian Ashnaei, Mohammad Mahdi Naghadian Moghaddam, Mohammad Shahrabi Farahani, Masood Soltanipur, Hossein Yarmohammadi

**Affiliations:** ^1^Student Research Committee, Faculty of Medicine, Shahed University, Tehran, Iran; ^2^School of Medicine, Islamic Azad University, Tehran Medical Branch, Tehran, Iran; ^3^School of Medicine, Qazvin University of Medical Sciences, Qazvin, Iran; ^4^Quality of Life Department, Breast Cancer Research Center, Motamed Cancer Institute, ACECR, Tehran, Iran; ^5^Ebne-sina Medical Center (EMC), Tehran, Iran

**Keywords:** 6-min walk test, 6MWT, cardiorespiratory fitness, lipedema, lymphedema, spirometry, VO_2_ peak

## Abstract

**Background:** One of the main challenges in managing lymphedema and lipedema is the lack of valid and reliable objective measures for diagnosis and follow-up. This study was aimed at gathering evidence regarding the objective measures of cardiorespiratory fitness (CRF) among these populations.

**Methods:** Scopus, PubMed, and Embase were searched for observational studies investigating the objective measures of CRF among individuals with lipedema and lymphedema. Both primary and secondary lymphedema were included. Different CRF measures reported by the included articles were determined, and the main outcomes regarding these measurements were extracted. The meta-analysis was performed to compare the pooled mean 6-min walk test (6MWT) between individuals with lower limb lymphedema and lipedema using STATA software (Version 17.0).

**Results:** Eight articles were included, and the majority of participants were female. Four distinct objective measures of CRF were reported among the included articles, including hemodynamic indices, spirometry indices, VO_2_ peak, and 6MWT. The mean VO_2_ peak was significantly lower among women with breast cancer–related lymphedema; however, there was no correlation between affected limb volumes and the VO_2_ peak. The meta-analysis revealed a lower mean 6MWT among individuals with lipedema compared to lymphedema (pooled difference: 37.71 [confidence interval (CI): 5.19–70.22], *p* value: 0.02, *I*^2^: 0%). Also, there was a significant relationship between 6MWT and subjective measures of CRF, such as the Short Form 36 (SF-36) physical function score, in one included article.

**Conclusion:** While limited evidence exists on the objective measures of CRF among individuals with lymphedema and lipedema, there might be a significant difference in 6MWT between these two groups.

## 1. Introduction

Lymphedema is a chronic condition characterized by an abnormal accumulation of lymphatic fluid in tissues, leading to swelling and discomfort. It commonly affects the limbs, particularly following surgery or radiation therapy for cancer; however, it also may occur due to genetic reasons [1, 2]. Living with a chronic condition like lymphedema can lead to feelings of frustration, anxiety, and depression, as individuals may experience changes in body image, self-esteem, and social interactions [3]. The visible swelling and discomfort associated with lymphedema can also contribute to negative emotions and self-perception. Individuals may also experience fatigue, pain, and limited range of motion in the affected limbs, hindering individuals' ability to perform everyday tasks such as walking, standing, or participating in physical activities [4, 5]. Furthermore, restricted mobility in the affected limbs can contribute to a sedentary lifestyle and decreased physical activity levels among individuals with lymphedema. This lack of movement and exercise can lead to the deconditioning of the cardiovascular and respiratory systems, further impacting their function and overall health.

Lipedema is a chronic condition characterized by an abnormal accumulation of fat cells in the legs, thighs, and buttocks, often leading to a disproportionate, asymmetrical swelling in these areas [6]. Individuals with lipedema often experience pain, tenderness, and easy bruising in the affected areas, as well as limitations in mobility and quality of life. Furthermore, these limitations can contribute to decreased physical activity and exercise tolerance, further impacting cardiorespiratory fitness (CRF) [7]. In addition, the psychosocial effects of living with a chronic condition like lipedema, such as changes in body image, self-esteem, and social interactions, can also influence individuals' motivation to engage in physical activities that promote cardiovascular and respiratory health [8].

One of the main neglects in lymphedema and lipedema management is the lack of objective measures for diagnosis and follow-up, rather than subjective evaluations, which may vary from one healthcare provider to another. Therefore, it is essential to provide objective measures of CRF for individuals with lymphedema and lipedema in order to improve their management outcomes. This systematic review was aimed at gathering evidence regarding objective measures of CRF in these two populations.

## 2. Methods

According to the Preferred Reporting Items for Systematic reviews and Meta-Analyses (PRISMA) guidelines, the method of this systematic review included the following steps [9]. The protocol of this systematic review is registered at PROSPERO and is accessible through the following link: CRD42024562347. Additionally, the PRISMA checklist for this study is available in Supporting Information 1: File S1.

### 2.1. Information Sources and Search Strategy

Search engines, including PubMed, Scopus, and Embase, were searched systematically in April 2024. The search was conducted based on combinations of relevant keywords as follows: lymphedema, lymphoedema, lipedema, lipoedema, cardiorespiratory fitness, cardiopulmonary fitness, cardiorespiratory capacity, and cardiopulmonary capacity. There was no restriction on the systematic search except that only articles in English were searched. Additionally, the references of included articles or related review papers were screened for possible eligible articles.

### 2.2. Eligibility Criteria and Selection Process

The screening was performed by two independent reviewers. In the case of any disagreement, the third senior author was asked for expert opinion. Observational studies that reported objective measures of CRF among individuals with lymphedema and lipedema were included. There was no restriction on the type of lymphedema, and both primary and secondary causes were included. Also, both upper and lower limb lymphedema (LLL) were eligible for this study. Articles that had reported subjective measures of CRF were excluded. For example, studies that had only reported results of CRF status based on questionnaires were not eligible. Objective measures of CRF included any conventional methods that are being used in clinical practice for cardiac fitness or pulmonary capacity assessment. These included but were not limited to hemodynamic measurement, spirometry, and a 6-min walk test (6MWT). Other types of articles, such as case reports, reviews, and interventional studies, were excluded. Also, articles that had not reported any statistical outcomes of CRF were ineligible. Moreover, animal studies and articles in languages other than English were not included.

### 2.3. Data Items and Collection Process

Relevant data from the selected studies were extracted by two investigators independently. These data included study design and primary aim, population, and main outcomes. The mean age, body mass index (BMI), and the percentage of females from each population were also gathered. Additionally, the correlation between CRF measures and lymphedema severity (determined based on volume or stage) was gathered.

### 2.4. Effect Measures and Synthesis Methods

The meta-analysis was performed for the difference in the mean 6MWT between individuals with lymphedema and lipedema. The mean ± standard deviation of the 6MWT was gathered from articles as the effect size of continuous outcomes. The heterogeneity was assessed based on the *I*^2^ > 40%, and *p* value > 0.05 was considered a low risk of heterogeneity. All analyses, including the forest plot, were performed based on the fixed model. STATA software (Version 17.0) was used for meta-analysis, and *p* values > 0.05 were considered significant.

### 2.5. Quality Assessment

The quality assessment for included articles was done based on the Joanna Briggs Institute (JBI) critical appraisal tool [10]. The JBI checklist is a well-known tool for evaluating the quality of included studies in systematic reviews. Different versions of the JBI checklist are available based on different study designs. For this systematic review, three checklists were used for cross-sectional, case-control, and cohort studies. The quality assessment was done by two authors independently, and the disagreements were thoroughly discussed and resolved accordingly. The quality was assessed based on different questions regarding the selection of participants, confounding factors, objectives, and exposure measurement. The answer to each question could be yes, no, unclear, or not applicable. Eventually, the percentage of yes answers was computed for each included article.

## 3. Results

**Figure 1:**
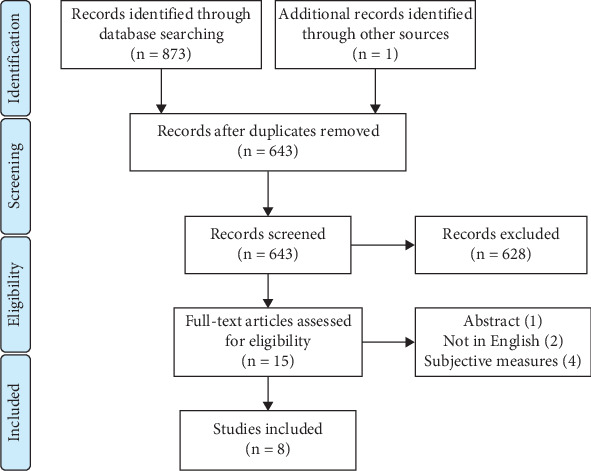
The PRISMA flowchart.

Eight observational studies were included [11–18] (Figure 1). Other than one longitudinal cohort study [12], all remaining articles were retrospective. Four articles were focused on breast cancer–related lymphedema (BCRL) [11, 15, 17, 18], while LLL was studied in two articles [12, 14]. Moreover, three studies investigated the CRF among individuals with lipedema [12–14]. More than 70% and 100% of included participants were female among lymphedema and lipedema populations, respectively (Table 1). The quality assessment resulted in favorable quality for the included articles, which is provided in Supporting Information 2: File S2 (Tables S1–S3). The majority of inadequate-quality articles were related to the identification of confounding factors and appropriate measures to reduce their effects.

Four objective measures of CRF were reported among the included articles: spirometry, VO_2_ peak, hemodynamic, and 6MWT. In one study, hemodynamic indices were significantly different among women with BCRL compared to healthy controls. While stroke volume, cardiac output, and left ventricular power were reduced among women with BCRL, systemic vascular resistance was significantly increased among this group. Also, different spirometry indices such as vital capacity, forced vital capacity, and forced expiratory volume in 1 s were reduced among women with BCRL compared to healthy controls [15]. However, in another study, no significant correlation was evident between BCRL severity (determined as the affected limb volume) and spirometry indices [11]. Moreover, an included study reported significantly lower VO_2_ peak among women with BCRL compared to breast cancer survivors (BCSs) without lymphedema. This difference was significant among the 40–49 age group. However, there was no relationship between variables related to BCRL, such as affected arm volume with VO_2_ peak based on the regression analysis [18].

**Figure 2:**
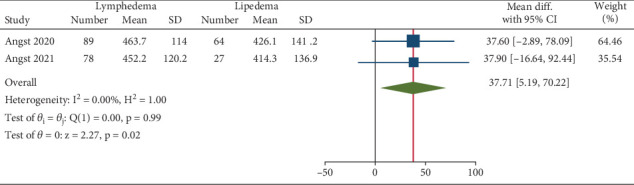
Meta-analysis of difference in mean 6MWT between individuals with lymphedema and lipedema.

The 6MWT was more frequently reported in included articles as a CRF objective measure. In two studies, the mean 6MWT was compared between individuals with LLL and lipedema [12, 14]. Although in each of these articles, the difference between the two groups was not significant, the meta-analysis revealed a significantly reduced 6MWT among individuals with lipedema compared to LLL (pooled difference: 37.71 [confidence interval (CI): 5.19–70.22], *p* value: 0.02). The heterogeneity of the meta-analysis was low based on the Higgins *I*^2^ statistical test (*I*^2^: 0%, *p* value > 0.05) (Figure 2). The mean 6MWT among individuals with lipedema was also compared with fibromyalgia and obesity in two other included studies. The mean values among the lipedema group were higher than those of the fibromyalgia group and lower than those of the obesity group; however, these differences were not statistically significant [13, 16]. Additionally, another study reported a lower mean 6MWT among women with BCRL compared to BCSs without BCRL, which was not significant [17]. Also, there was no significant correlation between BCRL severity (determined as the affected limb volume) and the 6MWT score [11]. Interestingly, in one study, the correlation between the 6MWT (as an objective measure of CRF) and the Short Form 36 (SF-36) physical function score (as a subjective measure of CRF) was investigated. Both individuals with LLL and lipedema were studied before and after rehabilitation sessions in this included article. The baseline mean 6MWT and SF-36 physical function scores were correlated in both groups of LLL and lipedema. However, changes in 6MWT and SF-36 physical function scores following the rehabilitation were not correlated in either of the groups [12].

## 4. Discussion

Based on the findings of this systematic review, there is limited evidence of objective measures for CRF evaluation among individuals with lymphedema and lipedema. Different populations were studied, which comprised women with lipedema, BCRL, and LLL. Four distinct objective measures of CRF were reported among the included articles: hemodynamic indices, spirometry indices, VO_2_ peak, and 6MWT. There was a significant difference in mean spirometry indices and VO_2_ peak among women with BCRL compared to healthy controls or BCSs without lymphedema, respectively. However, there was no correlation between lymphedema severity (determined as volume) with these measures. Although two included studies reported that the difference in mean 6MWT between individuals with lymphedema and lipedema was not significant, the meta-analysis revealed a statistically lower mean 6MWT among women with lipedema. At last, there was a significant relationship between 6MWT and subjective measures of CRF, such as the SF-36 physical function score.

The importance of these findings is in clinical implications both for practice and research. One of the main neglects in the field of lymphedema and lipedema management is the lack of valid and reliable objective measures for the diagnosis of these conditions. Currently, the diagnosis of lymphedema and lipedema relies heavily on subjective assessments, such as patient-reported symptoms and physical examinations by healthcare providers [2, 6]. While these methods can provide valuable information, they are not always reliable or consistent, leading to misdiagnosis or delayed diagnosis for some individuals. These subjective measures also are dependent on the opinion of the physicians, and therefore, they might vary based on their expertise [19]. Objective measures, such as imaging tests or lymphatic function assessments, can provide more concrete evidence of the presence and severity of these conditions, helping healthcare providers make more accurate diagnoses and develop appropriate treatment plans. Additionally, having objective measures for diagnosis can also help researchers better understand the underlying mechanisms of lymphedema and lipedema, leading to advancements in treatment options and management strategies [20, 21].

CRF refers to the ability of the cardiovascular and respiratory systems to deliver oxygen and nutrients to the tissues of the body, as well as remove carbon dioxide and other waste products. This is achieved through the coordinated efforts of the heart, lungs, blood vessels, and muscles, which work together to supply oxygen-rich blood to the body's cells and tissues [22, 23]. The CRF is essential for overall health and well-being, as it plays a crucial role in maintaining energy levels, endurance, and physical performance. CRF among BCSs can be altered due to a variety of factors related to cancer treatment and the disease itself. Firstly, treatments such as chemotherapy, radiation therapy, and surgery can have a significant impact on the cardiovascular and respiratory systems. Chemotherapy, for example, can cause damage to the heart muscle, leading to conditions such as cardiomyopathy or arrhythmias. Radiation therapy to the chest area can also damage the heart and lungs, affecting their function in the long term. Surgery, particularly if it involves the removal of lymph nodes in the armpit (axillary lymph node dissection), can lead to lymphedema and impaired lymphatic function, further impacting cardiorespiratory health [24]. Additionally, the physical and emotional impact of breast cancer and its treatments can also contribute to changes in CRF among survivors. Fatigue, muscle weakness, changes in body composition, and emotional distress can all affect a person's ability to exercise and perform daily activities, leading to deconditioning and decreased CRF [25]. Moreover, the increased body mass among individuals with obesity, lymphedema, and lipedema can lead to higher energy expenditure during physical activities, making it harder for the heart and lungs to supply oxygen and nutrients to the body's tissues. This can result in decreased CRF and endurance, as well as an increased risk of cardiovascular disease and respiratory complications. Furthermore, depression, anxiety, and low self-esteem can all impact a person's motivation to exercise and engage in physical activity, further exacerbating the negative effects on cardiorespiratory health [26].

The altered CRF among individuals with lymphedema and lipedema has important implications for their long-term health and quality of life. Decreased CRF is associated with an increased risk of cardiovascular disease, metabolic disorders, and overall mortality [23]. Therefore, it is crucial for healthcare providers to monitor and address these changes through appropriate interventions, such as exercise programs, pulmonary rehabilitation, and lifestyle modifications [27]. Addressing the altered CRF among individuals with lipedema and obesity is crucial for improving their overall health and quality of life. Healthcare providers should focus on implementing tailored exercise programs, weight management strategies, and lifestyle modifications to help individuals improve their CRF and reduce their risk of cardiovascular and respiratory complications [28, 29].

This article highlights current opportunities for clinical practice and also gaps for future research. While the majority of articles were focused on subjective measurements of CRF among individuals with lymphedema and lipedema, only limited studies investigated objective measurements such as spirometry or 6MWT. In a previous study, the lack of valid and reliable diagnostic approaches for these populations was considered a gap in this field. Moreover, there has been an increasing trend of recognition of musculoskeletal disorders among individuals with lymphedema and lipedema [30]. These musculoskeletal disorders are more likely to occur among these populations due to heaviness and anatomical disturbances, which eventually even lead to CRF alterations. This is of note that reducing pain, increasing range of motion, and improving CRF are among treatment goals and are linked to better quality of life outcomes [31]. Both lymphedema and lipedema are among neglected conditions with a significant burden imposed on affected individuals, specifically women. One interesting topic for future studies is investigating the beneficial effects of objective CRF measurements for timely lymphedema and lipedema management and reducing the occurrence of further complications with subsequent imposed costs [32].

There are some limitations to this systematic review that should be addressed. First of all, only English articles in certain search engines were reviewed. Therefore, this systematic review might not reflect all the existing evidence on the topic of CRF lymphedema and lipedema. Also, a few studies were included, and only two articles were eligible for the meta-analysis. This was mainly due to the lack of standard reporting of CRF among studies. While the results of the meta-analysis are limited to two articles from one research group, the pooled outcome was valuable. Each of these two included articles had concluded insignificant results; however, the pooled analysis demonstrated significantly lower 6MWT among individuals with lipedema compared to lymphedema. Additionally, both articles were of high quality, and the pooled heterogeneity was low; therefore, the results of this meta-analysis, even limited to two articles, still could be of value for practitioners in this field.

## 5. Conclusion

Hemodynamic indices, spirometry indices, VO_2_ peak, and 6MWT are among the reported objective measures of CRF among individuals with lymphedema and lipedema. Based on the meta-analysis, the mean 6MWT was significantly lower among women with lipedema compared to lymphedema. Future studies need to be more focused on the clinical application of objective measures of CRF for lymphedema and lipedema diagnosis and follow-up.

## Figures and Tables

**Table 1:** Characteristics of the included observational studies.

**Author, country (year)**	**Study design**	**Study aim**	**Population**	**Main outcome(s)**
Kutlu et al., Turkey (2023) [11]	Cross-sectional	Relationship between CRF and posture among BCSs	• 38 BCSs Age: 53, female: 100%, BMI: 27.3, 76% BCRL	• No significant correlation between spirometry and BCRL severity (affected limb volume): FEV1/FVC (*r*: −0.041), FEV1 (*r*: 0.309), MIP (*r*: −0.211), and MEP (*r*: −0.173) • No significant correlation between 6MWT and BCRL severity (affected limb volume): 6MWT (*r*: −0.302)
Thomas et al., Switzerland (2023) [12]	Longitudinal cohort	CRF among individuals with lipedema, lymphedema, and four other disorders before and after rehabilitation	• 27 lipedema Age: 50.8, female: 100%, BMI: 40.5 • 78 lymphedema Age: 59.4, female: 78.2%, BMI: 30.5 • 340 other disorders	• Correlation between baseline 6MWT and SF-36 physical function score: among lipedema (*r*: 0.791) and among lymphedema (*r*: 0.607) • Correlation between changes in 6MWT and SF-36 physical function score following the rehabilitation: among lipedema (*r*: 0.208) and among lymphedema (*r*: 0.129)
Angst et al., Switzerland (2021) [13]	Cross-sectional	CRF among individuals with lipedema and fibromyalgia	• 38 lipedema Age: 45.4, BMI: 33.7 • 77 fibromyalgia Age: 49.3, BMI: 27.4	• No significant difference in the mean 6MWT between the two groups
Angst et al., Switzerland (2020) [14]	Cross-sectional	Validity of CRF measurement among individuals with lymphedema and lipedema	• 96 lipedema Age: 46.7, female: 100%, BMI: 34.8 • 107 lymphedema Age: 56.4, female: 71%, BMI: 28.1	• No significant difference in the mean 6MWT between the two groups • Correlation between 6MWT and SF-36 physical function score (*r*: 0.8) only among the lipedema group
Odynets et al., Ukraine (2020) [15]	Case-control	CRF among women with BCRL compared to healthy controls	• 115 BCRL Age: 57.4, female: 100%, BMI: 25.9 • 50 healthy controls Age: 57.6, female: 100%, BMI: 26	• Significant difference of spirometry indices between the two groups: VC, FVC, FEV1, PEF, MEF_25_, MEF_50_, ERV, and MVV • No significant difference in the spirometry index of IRV between the two groups • Significant difference in hemodynamic indices between the two groups: stroke volume, stroke index, cardiac output, SVR, LV work, and LV power
van Esch-Smeenge, Damstra, and Hendrickx, the Netherlands (2017) [16]	Cross-sectional	CRF among individuals with lipedema and obesity	• 22 lipedema Age: 39.2, female: 100%, BMI: 33.5 • 22 obesity Age: 48.4, female: 100%, BMI: 35	• No significant difference in the mean 6MWT and 6MWT as percentage of normative value between the two groups
Galiano-Castillo et al., Spain (2016) [17]	Cross-sectional	CRF among BCSs with and without BCRL	• 87 BCSs 11.5% BCRL	• No significant difference in mean 6MWT between the two groups
Smoot et al., United States (2012) [18]	Cross-sectional	CRF among BCSs with and without BCRL	• 69 BCSs without BCRL Age: 55.1, female: 100%, BMI: 25.4 • 67 BCRL Age: 57.6, female: 100%, BMI: 26.7	• Significantly lower mean VO_2_ peak among BCRL group aged 40–49 years (*p* value: 0.01) • No significant regression between variables of BCRL and mean VO_2_ peak

Abbreviations: 6MWT, 6-min walk test; BCRL, breast cancer–related lymphedema; BCS, breast cancer survivor; BMI, body mass index; CRF, cardiorespiratory fitness; ERV, expiratory reserve volume; FEV, forced expiratory volume; FVC, forced vital capacity; IRV, inspiratory reserve volume; MEF, maximum expiratory flow; MIP, maximal inspiratory pressure; MVV, maximum voluntary ventilation; PEF, peak expiratory flow; SF-36, 36-item short form survey; VC, vital capacity.

## Data Availability

The data that support the findings of this study are available from the corresponding authors upon reasonable request.

## Nomenclature

6MWT: 6-min walk test
BCRL: breast cancer–related lymphedema
BCSs: breast cancer survivors
BMI: body mass index
CRF: cardiorespiratory fitness
JBI: Joanna Briggs Institute
LLL: lower limb lymphedema
PRISMA: Preferred Reporting Items for Systematic reviews and Meta-Analyses

## Supporting Information

Supporting Information 1 File S1: PRISMA checklist.

Supporting Information 2 File S2: Quality assessment.
