# Supplementary material for: Objective Assessment of the Cardiorespiratory Fitness Among Individuals With Lymphedema and Lipedema: A Systematic Review and Meta-Analysis
Source: Int J Vasc Med. 2025 Feb 13;2025:8627520. doi: 10.1155/ijvm/8627520 (PMC11842136; doi:10.1155/ijvm/8627520)
Supplement: Supporting Information 2 — File S2: Quality assessment. [file 8627520.f2.docx]

**Supplementary Table 1.** Results of Critical Appraisal Using JBI Analytical Cross-Sectional Studies Checklist

| **Study** | **Q1** | **Q2** | **Q3** | **Q4** | **Q5** | **Q6** | **Q7** | **Q8** | **Total (%) Yes** |
| --- | --- | --- | --- | --- | --- | --- | --- | --- | --- |
| Kutlu (2023) | N | Y | Y | Y | U | U | Y | Y | 62.5% |
| Angst (2021) | Y | Y | Y | Y | Y | U | Y | Y | 87.5% |
| Angst (2020) | Y | Y | Y | Y | Y | Y | Y | Y | 100% |
| van Esch-Smeenge (2017) | Y | N | Y | Y | Y | Y | Y | Y | 87.5% |
| Galiano-Castillo (2016) | Y | Y | Y | Y | U | U | Y | Y | 75% |
| Smoot (2012) | Y | Y | Y | Y | Y | Y | Y | Y | 100% |
| **Total (%) Yes** | **83.3%** | **83.3%** | **100%** | **100%** | **66.6%** | **50%** | **100%** | **100%** |  |

**JBI:** Joanna Briggs Institute, **N:** No, **NA:** Not Applicable, **U:** Unclear, **Y:** Yes.

**Critical appraisal questions:**

**Q1.** Were the criteria for inclusion in the sample clearly defined?

**Q2.** Were the study subjects and the setting described in detail?

**Q3.** Was the exposure measured in a valid and reliable way?

**Q4.** Were objective, standard criteria used for measurement of the condition?

**Q5.** Were confounding factors identified?

**Q6.** Were strategies to deal with confounding factors stated?

**Q7.** Were the outcomes measured in a valid and reliable way?

**Q8.** Was appropriate statistical analysis used?

**Supplementary Table 2.** Results of Critical Appraisal Using JBI Case-Control Studies Checklist

| **Study** | **Q1** | **Q2** | **Q3** | **Q4** | **Q5** | **Q6** | **Q7** | **Q8** | **Q9** | **Q10** | **Total (5) Yes** |
| --- | --- | --- | --- | --- | --- | --- | --- | --- | --- | --- | --- |
| Odynets (2020) | Y | Y | Y | Y | Y | N | U | Y | U | Y | 70% |

**JBI:** Joanna Briggs Institute, **N:** No, **NA:** Not Applicable, **U:** Unclear, **Y:** Yes.

**Critical appraisal questions:**

**Q1.** Were the groups comparable other than the presence of disease in cases or the absence of disease in controls?

**Q2.** Were cases and controls matched appropriately?

**Q3.** Were the same criteria used for identification of cases and controls?

**Q4.** Was exposure measured in a standard, valid, and reliable way?

**Q5.** Was exposure measured in the same way for cases and controls?

**Q6.** Were confounding factors identified?

**Q7.** Were strategies to deal with confounding factors stated?

**Q8.** Were outcomes assessed in a standard, valid, and reliable way for cases and controls?

**Q9.** Was the exposure period of interest long enough to be meaningful?

**Q10.** Was appropriate statistical analysis used?

**Supplementary Table 3.** Results of Critical Appraisal Using JBI Cohort Studies Checklist

| **Study** | **Q1** | **Q2** | **Q3** | **Q4** | **Q5** | **Q6** | **Q7** | **Q8** | **Q9** | **Q10** | **Q11** | **Total (%) Yes** |
| --- | --- | --- | --- | --- | --- | --- | --- | --- | --- | --- | --- | --- |
| Benz (2023) | Y | Y | Y | Y | U | Y | Y | Y | U | U | Y | 72.7% |

**JBI:** Joanna Briggs Institute, **N:** No, **NA:** Not Applicable, **U:** Unclear, **Y:** Yes.

**Critical appraisal questions:**

**Q1.** Were the 2 groups similar and recruited from the same population?

**Q2.** Were the exposures measured similarly to assign people to both exposed and unexposed groups?

**Q3.** Was the exposure measured in a valid and reliable way?

**Q4.** Were confounding factors identified?

**Q5.** Were strategies to deal with confounding factors stated?

**Q6.** Were the groups/participants free of the outcome at the start of the study (or at the moment of exposure)?

**Q7.** Were the outcomes measured in a valid and reliable way?

**Q8.** Was the follow-up time reported and sufficient to be long enough for outcomes to occur?

**Q9.** Was follow-up complete, and if not, were the reasons to loss to follow-up described and explored?

**Q10.** Were strategies to address incomplete follow-up utilized?

**Q11.** Was appropriate statistical analysis used?
